# Effectiveness of Intravitreal Ranibizumab in Nonvitrectomized and Vitrectomized Eyes with Diabetic Macular Edema: A Two-Year Retrospective Analysis

**DOI:** 10.1155/2020/2561251

**Published:** 2020-08-06

**Authors:** Ozgun Melike Gedar Totuk, Ayse Yagmur Kanra, Mohammed Nadim Bromand, Guler Kilic Tezanlayan, Sevil Ari Yaylalı, Irem Turkmen, Aylin Ardagil Akcakaya

**Affiliations:** ^1^Department of Ophthalmology, Bahcesehir University Faculty of Medicine, Kadikoy, Istanbul 34734, Turkey; ^2^Camlica Medicana, Goztepe Hospital, Uskudar, Istanbul 34767, Turkey; ^3^Corlu Dunya Goz Hospital, Corlu, Tekirdag 59860, Turkey; ^4^Gediz Public Hospital, Gediz, Kutahya 43600, Turkey; ^5^Medipol University, Camlica, Uskudar, Istanbul 34662, Turkey; ^6^Bahcesehir University Faculty of Medicine, Kadikoy, Istanbul 34734, Turkey; ^7^Atakoy Dunya Goz Hospital, Bakirkoy, Istanbul 34158, Turkey

## Abstract

The aim of this study was to compare the effectiveness of intravitreal ranibizumab (IVR) injections for the treatment of diabetic macular edema (DME) in eyes with and without previous vitrectomy. The medical records of 28 eyes (11 vitrectomized and 17 nonvitrectomized) of 28 patients (mean age, 59.0 ± 9.6 years; male to female ratio 1 : 1) who were diagnosed with DME and had received IVR treatment were reviewed retrospectively. The indications of vitrectomy in 11 vitrectomized eyes were intravitreal hemorrhage (*n* = 8) and epiretinal membrane (*n* = 3). The best-corrected visual acuity (BCVA), central macular thickness (CMT), and total macular volume (TMV) were measured at baseline and at months 6, 12, 18, and 24 of the follow-up. The number of IVR injections, the duration between diagnosis of DME and IVR injection, and the hemoglobin A1c (HbA1c) level at baseline were also recorded. Baseline demographics, HbA1c, BCVA, CMT, and TMV values were similar between two groups (*p* > 0.05). The duration between diagnosis of DME and IVR injections was similar in both groups (16 ± 5 months vs. 13 ± 4 months, respectively; *p*=0.11). IVR injection was performed 6.3 times in vitrectomized eyes and 6.1 times in nonvitrectomized eyes during the 24-month period (*p* > 0.05). The mean BCVA improved significantly during the 24-month period in both groups. The improvements in BCVA, in CMT, and in TMV were more significant at month 6 (*p*=0.036) group, at month 12 (*p*=0.013), at month 12 (*p*=0.021), and month 24 (*p*=0.021) in nonvitrectomized eyes, respectively, while there was no difference in improvements of BCVA, CMT, and TMV in vitrectomized group at each visit. Treatment effected by time in terms of BCVA, CMT, and TMV values in all groups (*p*=0.0004, *p* < 0.0001, *p* < 0.0001, respectively), not by time-group interaction and group (all *p* values >0.05). In conclusion, IVR treatment for DME is equally effective in both groups. However, the response to treatment is seen earlier in nonvitrectomized eyes compared to vitrectomized eyes.

## 1. Introduction

Diabetic macular edema (DME) is the most common cause of visual impairment in patients with diabetic retinopathy with a prevalence of 2.7%–11% [1].

The ophthalmic treatment of DME includes intravitreal antivascular endothelial growth factor (anti-VEGF) drug injections, intravitreal corticosteroid injections, focal/grid argon laser photocoagulation, subthreshold micropulse diode laser photocoagulation, and vitrectomy. Since 2010, anti-VEGF drug injections have become standard therapy for DME with the proven benefit of improved visual acuity [1–6].

Vitrectomy, as treatment for DME, was first introduced for eyes with proliferative diabetic retinopathy (PDR), unresolving vitreous hemorrhage, significant vitreomacular traction commonly associated with shallow traction macular detachment, and persistent DME despite previous focal laser or intravitreal injections. Vitrectomy has recently been studied as potential primary therapy in eyes with more severe edema and greater visual acuity loss at presentation [7, 8].

There is a controversy regarding the effects of vitrectomy on the diffusion and clearance of intravitreal anti-VEGF drugs for DME. Some animal studies have shown this clearance to be faster, while others have failed to show any pharmacokinetic changes of intravitreal drugs after vitrectomy [7–9]. In theory, faster clearance of intravitreal drugs could mean decreased effectiveness in vitrectomized eyes [9, 10].

Intravitreal ranibizumab (IVR), an anti-VEGF drug, has been shown to be an effective treatment for DME, providing a significant improvement in best-corrected visual acuity (BCVA) and in anatomic outcomes [3, 10–12]. There are limited data on the comparison of the efficacy of IVR in vitrectomized and nonvitrectomized eyes with DME. Chen et al. [3] showed that IVR was effective in both vitrectomized and nonvitrectomized eyes with DME in a 6-month follow-up. They reported that greater anatomical and functional improvements were obtained in nonvitrectomized patients than in vitrectomized cases. However, these findings only show the short-term outcome of the treatment. Bressler et al. [13] reported no benefical effect of vitrectomy in eyes with severe baseline diabetic retinopathy treated with anti-VEGF for 36 months.

The aim of this study is to compare the long-term effectiveness of IVR for treatment of DME in vitrectomized and nonvitrectomized eyes.

## 2. Materials and Methods

### 2.1. Study Design and Population

In this retrospective comparative study, we reviewed the medical records of 11 vitrectomized eyes of 11 patients (mean age, 55.0 ± 10.0 years; male to female ratio, 6 : 5) and 17 nonvitrectomized eyes of 17 patients (mean age, 62.0 ± 9.0 years; male to female ratio 8 : 9) with severe nonproliferative diabetic retinopathy or proliferative diabetic retinopathy who received naïve IVR injections and were treated by panretinal photocoagulation previously (Table 1). They were followed up for at least 24 months between April 2013 and December 2017 at Atakoy Dunyagoz Hospital.

Patients with alterations that could prevent improvement in BCVA (the presence of apparent retinal pigment epithelium (RPE) atrophy or proliferative diabetic fibrovascular membranes at or near the macula and the presence of diabetic or glaucomatous optic atrophy) in medical records, active intraocular inflammation or infection in one or both eyes, uncontrolled or neovascular glaucoma, prior treatment with intravitreal or periocular pharmacologic injections in the studied eye within a 3-month period before the IVR injections, panretinal laser photocoagulation within 6 months or macular focal/grid laser photocoagulation in the studied eye within a 3-month period before the beginning of the IVR injections, and previous major surgeries such as cataract extraction or steroid injections within the previous 3 months or during the course of the IVR injections were excluded.

### 2.2. Ethical Approval

This study was approved by the Institutional Ethics Committee of Bahcesehir University (Mar/20th/2019; 2019/06/01) and conducted in accordance with the latest version of the Declaration of Helsinki. According to the Regulation on Clinical Studies of Drugs and Biological Products in Turkey (no: 29474), updated on September 13, 2015, retrospective studies are not subject to the requirement of informed consent of patients. The Institutional Ethics Committee of Bahcesehir University, which operates in accordance with this regulation, waived the requirement of informed consent for this study. All patients with vitrectomized eyes were informed about the risks and benefits of vitrectomy before surgery, and written consent was obtained after a thorough explanation of the procedure. The potential risks and benefits of IVR injections were also discussed extensively with all the patients. All patients gave written informed consent for IVR injections.

### 2.3. Study Procedures

All patients underwent a comprehensive clinical assessment and ophthalmologic examination including measurement of the BCVA and indirect and contact lens slit lamp fundoscopic examination. The BCVA was measured with a standard Snellen chart and converted to the logarithm of the minimum angle of resolution (logMAR) units. Spectral domain or swept source optical coherence tomography (OCT) (Topcon 3D OCT-2000, Tokyo, Japan) was used to examine the central macular thickness (CMT) and total macular volume (TMV) of all eyes before surgery at baseline and at months 6, 12, 18, and 24 of the follow-up. In OCT retinal thickness measurement (the distance between the inner surface of RPE and the inner surface of the neurosensory retina), a 3D model of the retina was computed and retinal volumes (RVs) were assessed for each of the nine subfields using the inner, intermediate, and outer rings (with diameters of 1 mm, 2.22 mm, and 3.45 mm, respectively) as defined by the Early Treatment Diabetic Retinopathy Study (ETDRS) [12]. CMT was defined as the average thickness of the macula in the central 1 mm ETDRS grid. TMV was calculated by summation of all the volumes obtained in the ETDRS subfields.

The number of intravitreal injections, the duration between the diagnosis of DME and IVR injections, and hemoglobin A1c (HbA1c) levels at baseline were also assessed.

### 2.4. Vitrectomy Surgery

Vitrectomy was performed at least 3 months prior to the start of IVR treatment.

The indications for pars plana vitrectomy (PPV) were intravitreal hemorrhage (*n* = 8) and epiretinal membrane due to chronic diabetic macular edema (*n* = 3). The internal limiting membrane (ILM) was peeled in patients operated on for epiretinal membrane. Before the appearance of DME, the macula was flat in all vitrectomized patients after surgery.

### 2.5. Intravitreal Ranibizumab Treatment

The indications for anti-VEGF treatment for eyes with DME were CMT of more than 300 *μ*m determined by spectral-domain OCT and/or decimal BCVA less than 0.7. The intravitreal dose of ranibizumab was 0.5 mg/0.05 ml. All patients were treated with a PRN regimen from the beginning with monthly follow-ups. A reduction of >10% in CMT was defined as an anatomical improvement considering interexamination measurement bias. Retreatment criteria included persistence of submacular fluid, intraretinal cysts, or CMT of more than 300 *μ*m.

### 2.6. Statistical Analysis

Continuous variables were expressed as mean and standard deviation (SD) and categorical variables as number and percentage. The distribution of the continuous variables was evaluated with histograms and Q-Q plots along with Shapiro–Wilk's test. Age, HbA1c, and time to IVR values were normally distributed, thus, were compared between groups using Student's *t*-test. Categorical data were compared using the chi-square tests when needed. A repeated measure analysis of variance (ANOVA) test was conducted to assess the effect of treatment on BCVA, CMT, and TMV scores for each study group. Effect of treatment between two groups was compared with the mixed-design (split-plot) ANOVA test with one within-subject factor (time, 5 levels) and one between-subject factor (group, 2 levels). When time, group, or interaction effects were significant, Bonferroni correction was used to examine pairwise comparisons at each time point. Mauchly's test of sphericity was used to test the variances of the differences between repeated measurements at different time points. When the assumption of sphericity was violated, the Greenhouse–Geisser correction was used. The assumption of equality of covariance matrices was tested by Box's test, and the homogeneity of variance was tested using Levene's test. A 2-tailed *p* value of <0.05 was considered statistically significant. All statistical analyses were performed using the IBM SPSS software (IBM SPSS Statistics for Windows, Version 21.0., Armonk, NY, IBM Corp.).

## 3. Results and Discussion

A total of 28 patients (mean age: 59.0 ± 9.6, female: 50%) were included, 17 in the nonvitrectomized group and 11 in the vitrectomized group. The two groups were similar with respect to age and gender distribution, baseline HbA1c, BCVA, CMT, and TMV values (Tables 1 and 2, all *p* > 0.05). In the vitrectomized group, 7 (64%) and 4 (36%) eyes were pseudophakic and phakic, respectively. The corresponding figure for the nonvitrectomized group was 10 eyes (59%) and 7 eyes (41%), respectively. Cataract formation that needs phacoemulsification surgery was not observed in phacic eyes during the 24-month follow-up period. Two patients in the vitrectomized group and three patients in the nonvitrectomized group had received focal argon laser photocoagulation treatment at least 3 months before IVR treatment. Baseline demographics and characteristics of the patients are shown in Tables 1 and 2.

**Figure 1:**
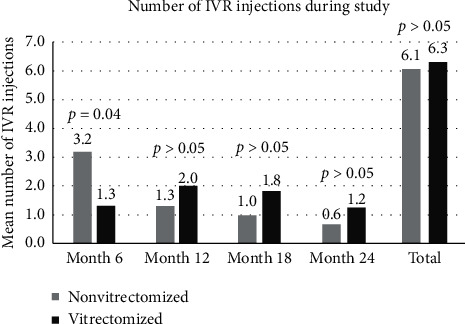
The mean number of intravitreal ranibizumab (IVR) injections given to the study groups during 24 months of follow-up. *p* values indicate the outcome of statistical testing for the comparison between groups for the corresponding time of evaluation. IVR injection was performed 6.3 times in vitrectomized eyes and 6.1 times in nonvitrectomized eyes during the 24 months of follow-up (*p* > 0.05). The number of IVR injections within the first 6 months was significantly higher in the nonvitrectomized group than in the vitrectomized group (3.2 vs. 1.3, *p*=0.04).

The number of IVR injections in vitrectomized and nonvitrectomized groups is shown in Figure 1.

**Figure 2:**
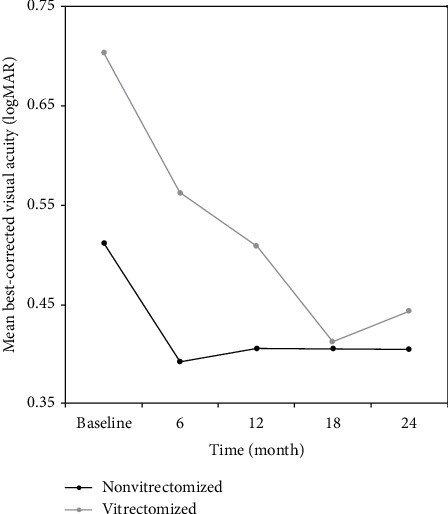
Changes in the mean visual acuity (in logMAR) of the study groups during 24 months of follow-up. BCVA indicates best-corrected visual acuity. Post hoc tests using the Bonferroni correction revealed that IVR treatment elicited a significant improvement in BCVA values from baseline to 6th month of treatment (0.51 ± 0.26 vs. 0.39 ± 0.26, *p*=0.036) in the nonvitrectomized group.

**Figure 3:**
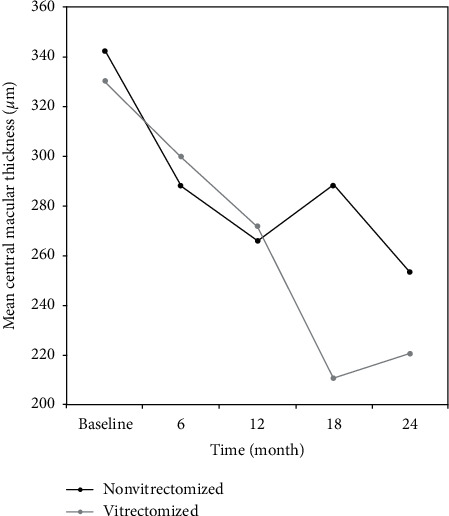
Changes in the mean central macular thickness (µ) of the study groups during 24 months of follow-up. CMT indicates central macular thickness. For CMT values, the reduction from baseline to the 12th month of the treatment was significant in the nonvitrectomized group (342 ± 98 vs. 266 ± 111, *p*=0.013).

**Figure 4:**
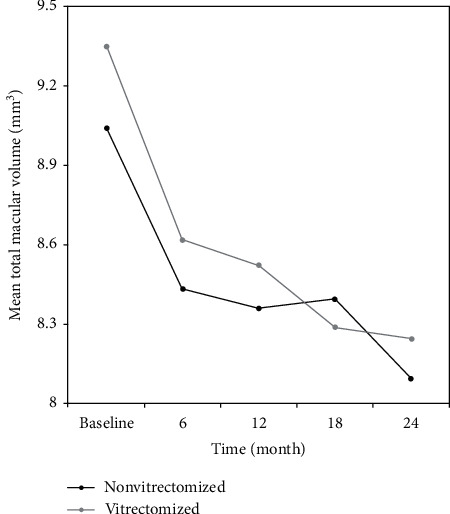
Changes in the mean total macular volume (TMV, mm^3^) of the study groups during 24 months of follow-up. There was a significant reduction in TMV values from baseline to the 12th month (9.04 ± 1.28 vs. 8.36 ± 1.10, *p*=0.021) and from baseline to the 24th month (9.04 ± 1.28 vs. 8.01 ± 0.79, *p*=0.021) in the nonvitrectomized group.

BVCA, CMT, and TMV values of nonvitrectomized and vitrectomized eyes at each time point are presented in Tables 3 and 4. Intravitreal ranibizumab treatment had a statistically significant effect on BCVA, CMT, and TMV values in both nonvitrectomized (all *p* values <0.05, Table 3, Figures 2–4) and vitrectomized eyes (all *p* values <0.05, Table 4, Figures 2–4).

The results of the mixed-design (split-plot) ANOVA test comparing the effect of the treatment on BCVA, CMT, and TMV values in nonvitrectomized and vitrectomized groups are shown in Table 5.

## 4. Discussion

PPV improves visual acuity by reducing macular thickness in patients with DME [13–15]. It also reduces retinal ischemia by allowing better oxygenation of the retina [16–18] and has the potential to increase the diffusion and clearance of intravitreal anti-VEGF drugs used for DME [9, 10]. It is, therefore, clinically important to know whether vitreoretinal surgery alters the anatomical and visual effects of anti-VEGFs in patients with DME. In this study, we compared the effectiveness of IVR injections for the treatment of DME in eyes with and without previous vitrectomy and found that although the functional response to treatment is seen earlier in nonvitrectomized eyes, IVR is an equally effective treatment for DME in both vitrectomized and nonvitrectomized eyes.

Ahn et al. [19] compared the concentration and elimination of IVR in vitrectomized and nonvitrectomized rabbit eyes and showed that the concentration of ranibizumab in both groups was not significantly different at 30 days after intravitreal injection. In contrast, Lee et al. [20] showed that the half-life of human recombinant VEGF (hVEGF) in the vitreous cavity was 10 times shorter in vitrectomized eyes, and hVEGF clearance increased after vitrectomy. However, the mechanisms of elimination of ranibizumab and other drugs in the vitreous cavity are not fully understood [19, 21–23]. The high molecular weights of the drugs are an important factor in the human and rabbit vitreous cavity, which affect the half-life of the drug. For example, the half-life of low molecular weight drugs such as amikacin or ceftazidime (molecular weight < 1,000) is between 2 and 10 hours, while the half-life of antibody fragments such as ranibizumab (molecular weight ≈ 48,000) is 2-3 days [24, 25]. Unlike similar molecular weight substances, the half-life of hVEGF (molecular weight 42,000) in the vitreous cavity was less than 3 hours [20]. This suggests that rapid cleaning mechanisms exist to regulate the levels of the hVEGF molecule in the vitreous cavity. Supporting this, in our study, no significant difference was found between vitrectomized and nonvitrectomized eyes at the end of the 2 years for BCVA, CMT, TMV, and total number of IVR injections.

In a recent study, Chen et al. [3] retrospectively compared the efficacy of IVR in vitrectomized and nonvitrectomized eyes in 148 patients with DME for up to 6 months. They reported significantly improved BCVA and central foveal thickness in nonvitrectomized eyes than in vitrectomized eyes [3]. Koyanagi et al. [4] compared the efficacy of IVR in 10 vitrectomized and 15 nonvitrectomized eyes and reported no significant differences in the mean changes of BCVA and CMT between both groups at 6 months. Bressler et al. [13] prospectively compared anti-VEGF treatment in 25 vitrectomized eyes with 335 nonvitrectomized eyes during 36 months. They found that the vitrectomized group had slower response in CMT during the first year, but BCVA improvement was similar in both groups. Our short-term (6-month) BCVA findings in favor of nonvitrectomized eyes were in line with these reports. We found that, in the vitrectomized group, BCVA, CMT, and TMV significantly improved at each time point of visit throughout the 24-month follow-up. Improvement in all parameters was recorded earlier in the nonvitrectomized group than in the vitrectomized group, BCVA significantly improved at the month 6 visit, CMT at the month 12 visit, and TMV at the month 12 and 24 visits.

Similar to our findings, Bressler et al. [13, 26] demonstrated that a comparable improvement was achieved in both vitrectomized and nonvitrectomized eyes treated with ranibizumab for DME at long-term follow-up. The slower response in the vitrectomized group was most probably due to higher number of IVR injections up to month 6 in nonvitrectomized eyes compared to vitrectomized eyes. Although response was obtained slowly in vitrectomized patients, there was no difference between groups in the long run. Also, the effect of time-group interaction and the group did not show any difference in all patients. We also recorded a significant improvement in BCVA values from baseline to the 18th and 24th month visits, CMT values from baseline to the 12th, 18^th^, and 24th month visits, and TMV values from baseline to the 6th, 12th, 18^th^, and 24th month visits in nonvitrectomized eyes compared to vitrectomized eyes. The late-term time-dependent improvements in both groups suggest the need of a long follow-up period of the patients treated with anti-VEGF injections for DME.

Chen et al. [3] reported that the number of IVR injections was significantly less in nonvitrectomized than in vitrectomized eyes (4.1 ± 0.6 vs. 5.1 ± 0.7, respectively; *p* < 0.001) during a 6-month period. In the study by Koyanagi et al. [4], the number of IVR injections during the 6-month period was similar in both nonvitrectomized and vitrectomized eyes (4.5 ± 1.2 vs. 4.9 ± 1.3, respectively; *p*=0.484). Bressler et al. [13] reported that cumulative number of IVR injections during the 6-month treatment period and at the end of 3-year follow-up was similar between the nonvitrectomized and the vitrectomized groups (5.2 ± 1.0 vs. 5.4 ± 0.9, respectively at 6 months; 14.3 ± 7.4 in both groups at 36 months). In our study, the number of IVR injections until month 6 was significantly higher in nonvitrectomized eyes than that in vitrectomized eyes (3.2 vs. 1.3, respectively; *p*=0.04). However, it is important to note that total number of IVR injections during 24 months of study duration was not statistically different between nonvitrectomized and vitrectomized eyes (6.1 vs. 6.3, respectively).

PPV has considerable consequences for the retinal physiology and for the pharmacokinetic properties of intravitreal anti-VEGF agents. In addition to relieving tangential and vitreomacular traction, PPV has advantages of decreasing the amount of VEGF and proinflammatory cytokines and improving the retinal perfusion and oxygen supply [27–33]. Christoforidis et al. [9] reported that intravitreal clearance of bevacizumab and ranibizumab was faster after pars plana lensectomy or PPV compared with nonsurgical eyes in a rabbit model. This finding suggests that PPV might lower the potency of intravitreal anti-VEGF injections. In our study, we found that cumulative number of anti-VEGF injections had no effect on the clinical outcomes of both vitrectomized and nonvitrectomized patients. We conclude that benefical effect of PPV on the retina such as removing depot of VEGF and proinflammatory cytokines and improving retinal oxygen delivery and perfusion may counterbalance the increased clearance rate of intravitreally placed anti-VEGF agents after PPV.

The main limitation of our study is its small sample size which precludes us from reaching more definitive conclusions on the long-term effectiveness of IVR in vitrectomized and nonvitrectomized eyes in patients with DME. Furthermore, the study has typical limitations of retrospective design such as inability to control the treatment schedule in all patients which makes the study groups not completely comparable. Despite all its limitations, this study with up to a 24-month follow-up compares the effectiveness of IVR in vitrectomized and nonvitrectomized eyes with similar baseline characteristics. Further large-scale prospective and long-term studies are needed to confirm our findings.

## 5. Conclusions

In conclusion, although the functional response to treatment was obtained later in vitrectomized eyes compared to nonvitrectomized eyes, IVR injection treatment for DME is equally effective in both vitrectomized and nonvitrectomized eyes in the long term.

## Figures and Tables

**Table 1:** Baseline characteristics of the study groups.

	Total (*n* = 28)	Nonvitrectomized (*n* = 17)	Vitrectomized (*n* = 11)	*p* value
Age (year)	59.0 ± 9.6	62.0 ± 9.0	55.0 ± 10.0	0.06^*∗*^
Female	14 (50.0%)	9 (52.9%)	5 (45.5%)	0.699^*∗∗*^
Hemoglobin A1c	7.6 ± 1.0	7.6 ± 1.1	7.7 ± 0.8	0.8^*∗*^
Time to IVR (month)†	15 ± 5	16 ± 5	13 ± 4	0.11^*∗*^

Continuous variables are presented as mean and standard deviation, and categorical variables are presented as number and percentage. ^†^Duration from diagnosis of DM to IVR injections, ^*∗*^Student's *t*-test, and ^*∗∗*^chi-square test. DM indicates diabetes mellitus; IVR, intravitreal ranibizumab.

**Table 2:** Baseline ocular measurements of the study groups.

	Total (*n* = 28 eyes)	Nonvitrectomized (*n* = 17 eyes)	Vitrectomized (*n* = 11 eyes)	*p*
BCVA (logMAR)	0.59 ± 0.29	0.51 ± 0.26	0.70 ± 0.3	0.084^*∗*^
CMT (*μ*m)	337.57 ± 95.53	342.41 ± 98.31	330.09 ± 95.26	0.746^*∗*^
TMV (mm³)	9.16 ± 1.36	9.04 ± 1.28	9.35 ± 1.52	0.568^*∗*^

Data are presented as mean and standard deviation. BCVA was measured with a standard Snellen chart and converted to the logarithm of the minimum angle of resolution (logMAR) units. BCVA indicates best-corrected visual acuity; CMT, central macular thickness; TMV, total macular volume. ^*∗*^Student's *t*-test.

**Table 3:** Effect of treatment on BCVA, CMT, and TMV values in the nonvitrectomized group (*n* = 17).

	Mean ± SD	*F* value	*p* value^*∗*^	Partial eta^2^
BCVA (logMAR)				
Baseline	0.51 ± 0.26	4.645	0.002	0.225
6^th^ month	0.39 ± 0.26			
12^th^ month	0.41 ± 0.25			
18^th^ month	0.41 ± 0.24			
24^th^ month	0.40 ± 0.22			

CMT (*μ*m)				
Baseline	342 ± 98	4.648	0.002	0.225
6^th^ month	288 ± 93			
12^th^ month	266 ± 111			
18^th^ month	288 ± 97			
24^th^ month	253 ± 97			

TMV (mm^3^)				
Baseline	9.04 ± 1.28	7.363	0.001	0.315
6^th^ month	8.43 ± 1.04			
12^th^ month	8.36 ± 1.10			
18^th^ month	8.40 ± 0.95			
24^th^ month	8.01 ± 0.79			

BCVA was measured with a standard Snellen chart and converted to the logarithm of the minimum angle of resolution (logMAR) units. BCVA indicates best-corrected visual acuity; CMT, central macular thickness; SD, standard deviation; TMV, total macular volume. ^*∗*^Repeated measure ANOVA. Other than BCVA values from baseline to the 6th month of treatment, CMT values from baseline to the 12th month of the treatment, and TMV values from baseline to the 12th month and from baseline to the 24th month of the treatment, pairwise comparisons for BCVA, CMT, and TMV values were not significant in the nonvitrectomized group.

**Table 4:** Effect of treatment on BCVA, CMT, and TMV values in vitrectomized eyes (*n* = 11).

	Mean ± SD	*F* value	*p* value^*∗*^	Partial eta^2^
BCVA (logMAR)				
Baseline	0.70 ± 0.30	4.304	0.038	0.301
6^th^ month	0.56 ± 0.28			
12^th^ month	0.51 ± 0.32			
18^th^ month	0.41 ± 0.28			
24^th^ month	0.44 ± 0.24			

CMT (*μ*m)				
Baseline	330 ± 95	6.122	0.005	0.380
6^th^ month	300 ± 106			
12^th^ month	272 ± 85			
18^th^ month	211 ± 96			
24^th^ month	221 ± 117			

TMV (mm^3^)				
Baseline	9.35 ± 1.52	4.467	0.030	0.309
6^th^ month	8.62 ± 0.86			
12^th^ month	8.52 ± 0.91			
18^th^ month	8.29 ± 1.08			
24^th^ month	8.25 ± 1.47			

BCVA was measured with a standard Snellen chart and converted to the logarithm of the minimum angle of resolution (logMAR) units. BCVA indicates best-corrected visual acuity; CMT, central macular thickness; SD, standard deviation; TMV, total macular volume. ^*∗*^Repeated measure ANOVA. None of the pairwise comparisons for BCVA, CMT, and TMV values were significant in the vitrectomized group.

**Table 5:** Comparison of the effect of intravitreal ranibizumab on BCVA, CMT, and TMV values in nonvitrectomized and vitrectomized eyes.

	*F* value	*p* value^*∗*^	Partial eta^2^
BCVA (logMAR)			
Main effect of time	8.999	0.0004	0.257
Main effect of time-group interaction	2.319	0.108	0.082
Main effect of group	1.283	0.268	0.047

CMT (*μ*m)			
Main effect of time	9.419	<0.0001	0.266
Main effect of time-group interaction	1.963	0.127	0.070
Main effect of group	0.458	0.505	0.017

TMV (mm^3^)			
Main effect of time	11.420	<0.0001	0.305
Main effect of time-group interaction	0.429	0.685	0.016
Main effect of group	0.138	0.714	0.005

BCVA indicates best-corrected visual acuity; CMT, central macular thickness; TMV, total macular volume. ^*∗*^Mixed-design ANOVA with one within-subject factor (time: 5 levels) and one between-subject factor (group: 2 levels). The main effect of time on BCVA (*F*(2.032, 52.827) = 8.999, *p* = 0.0004, partial eta^2^ = 0.257), CMT (*F*(2.973, 77.295) = 9.419, *p* < 0.0001, partial eta^2^ = 0.266), and TMV (*F*(2.345, 60.957) = 11.420, *p* < 0.0001, partial eta^2^ = 0.305) was statistically significant. The main effect of time-group interaction and the group was not statistically significant (all *p* values >0.05, Table 5). A post hoc pairwise comparison showed a significant improvement in BCVA values from baseline to the 18th and 24th months of treatment (*p*=0.004 and *p*=0.009, respectively). There was a significant reduction in CMT values from baseline to the 12th, 18^th^, and 24th months of treatment (*p*=0.005, *p*=0.001, and *p*=0.002, respectively). The reduction in TMV values was significant from baseline to the 6th, 12th, 18^th^, and 24th months of treatment (*p*=0.04, *p*=0.01, *p*=0.004, and *p*=0.001, respectively). All other pairwise comparisons for BCVA, CMT, and TMV values were not statistically significant.

## Data Availability

All necessary data are included within the manuscript.
